# Sequencing of the Pituitary Transcriptome after GnRH Treatment Uncovers the Involvement of lncRNA-m23b/miR-23b-3p/CAMK2D in FSH Synthesis and Secretion

**DOI:** 10.3390/genes14040846

**Published:** 2023-03-31

**Authors:** Tian Wang, Guokun Zhao, Song Yu, Yi Zheng, Haixiang Guo, Haoqi Wang, Peisen Zhao, Wenyin Xie, Wenzhi Ren, Bao Yuan

**Affiliations:** 1 Department of Laboratory Animals, College of Animal Sciences, Jilin University, Changchun 130062, China; 2 Jilin Provincial Key Laboratory of Animal Model, Jilin University, Changchun 130062, China

**Keywords:** GnRH, rat adenohypophysis cells, lncRNA-m23b, miR-23b-3p, CAMK2D, FSH, animal reproduction

## Abstract

The pituitary gland is a key participant in the hypothalamic–pituitary–gonadal axis, as it secretes a variety of hormones and plays an important role in mammalian reproduction. Gonadotrophin-releasing hormone(GnRH) signaling molecules can bind to GnRH receptors on the surfaces of adenohypophysis gonadotropin cells and regulate the expression of follicle-stimulating hormone(FSH) and luteinizing hormone(LH) through various pathways. An increasing number of studies have shown that noncoding RNAs mediate the regulation of GnRH signaling molecules in the adenohypophysis. However, the expression changes and underlying mechanisms of genes and noncoding RNAs in the adenohypophysis under the action of GnRH remain unclear. In the present study, we performed RNA sequencing (RNA-seq) of the rat adenohypophysis before and after GnRH treatment to identify differentially expressed mRNAs, lncRNAs, and miRNAs. We found 385 mRNAs, 704 lncRNAs, and 20 miRNAs that were significantly differentially expressed in the rat adenohypophysis. Then, we used a software to predict the regulatory roles of lncRNAs as molecular sponges that compete with mRNAs to bind miRNAs, and construct a GnRH-mediated ceRNA regulatory network. Finally, we enriched the differentially expressed mRNAs, lncRNA target genes, and ceRNA regulatory networks to analyze their potential roles. Based on the sequencing results, we verified that GnRH could affect FSH synthesis and secretion by promoting the competitive binding of lncRNA-m23b to miR-23b-3p to regulate the expression of Calcium/Calmodulin Dependent Protein Kinase II Delta(CAMK2D). Our findings provide strong data to support exploration of the physiological processes in the rat adenohypophysis under the action of GnRH. Furthermore, our profile of lncRNA expression in the rat adenohypophysis provides a theoretical basis for research on the roles of lncRNAs in the adenohypophysis.

## 1. Introduction

The pituitary gland is an important part of the mammalian endocrine and reproductive systems. It is located in the sella turcica at the base of the skull and includes the anterior, middle, and posterior lobes [1]. The posterior pituitary, also known as the neurohypophysis, mainly stores and secretes vasopressin (VP) and oxytocin (OT), which are synthesized by neurons in the hypothalamus [2]. The anterior and middle pituitary glands together form the adenohypophysis, which secretes thyroid-stimulating hormone (TSH), growth hormone (GH), follicle-stimulating hormone (FSH), luteinizing hormone (LH), prolactin (PRL), and other hormones [3]. The proportions of different cell types in the adenohypophysis are affected by various hormones in the body [4]. However, GH-secreting cells usually account for approximately 50% of cells and are the most abundant cells in the pituitary gland; the remaining four cell types have similar proportions of approximately 10–20% each [5]. The gonadotropins that secrete LH and FSH are the last cells to differentiate among anterior adenohypophysis cells, which reflects the importance of gonadotropins to pituitary function [6]. Gonadotropins include FSH and LH, which consist of a specific β subunit and a common α subunit. *Cga* encodes the α subunit [7], and *Lhb* and *Fshb* encode the β subunit [8]. In addition, studies have shown that gonadotropin-releasing hormone (GnRH) secreted by the hypothalamus can act on the pituitary to regulate gonadotropin synthesis [9,10].

GnRH is a neurohormone that is synthesized and secreted by hypothalamic neurons (GnRH neurons). It acts on pituitary gonadotropins through the vascular portal system and promotes the release of FSH and LH [11]. GnRH can bind to the GnRH receptor (GnRHR), located on the surfaces of adenohypophysis gonadotropins, to induce the *Cga* promoter, *Fshb*, and *Lhb* to promote gene transcription [12,13,14]. GnRH signaling molecules have been found to regulate the expression of *Fshb* and *Lhb* through the cAMP, CAMK II, and MAPK signaling pathways, which are important for the accurate regulation of GnRH [15,16,17]. Genes such as CAMK2D, MAPK3, and CAMK2A are also involved in the GnRH signaling pathway [18]. Ca^2+^/CAMK II plays a key role in the transmission of GnRH signals from the plasma membrane to the rat *Fshb* and *Lhb* subunit genes [19]. However, critical validation of how GnRH activates Ca^2+^/CAMK II and thus affects FSH synthesis and secretion is lacking.

GnRH and its analogs have an important application value in animal production. For example, immunocastration by inoculation with GnRH can replace surgical castration to promote animal development [20]. Treatments with a certain concentration of GnRH can effectively increase the concentration of progesterone in dairy cows, which is conducive to the establishment and maintenance of pregnancy [21]. However, the effects of GnRH administration vary among individuals, and the molecular mechanism of action of GnRH has not been completely clarified. The rat is a common experimental animal model for research on the effect of GnRH on the pituitary gland. Various studies have used rat experimental animal models to explore the expression changes of gonadotropins in the adenohypophysis after a GnRH treatment [22,23,24].

Previous studies have reported that GnRH can regulate the expression of noncoding RNAs (ncRNAs) in mammals. For example, in rats with liver fibrosis, GnRH treatment can significantly upregulate the expression of miR-200b and liver fibrosis markers in the liver [25]. GnRH inhibits miR-7 expression in the mouse hypothalamic–pituitary–ovarian axis [26]. Related studies have reported that GnRH treatment can alter microRNA (miRNA) expression profiles in mouse LβT2 gonadotropic cells and porcine anterior pituitary cells [9,27]. Our previous study also revealed that after GnRH treatment, the levels of circRNAs in the rat adenohypophysis are changed, and 14 circRNAs are significantly differentially expressed [22]. NcRNAs in the pituitary can play important roles. CircAkap17b competitively binds miR-7 family members to regulate FSH synthesis and secretion in rat adenohypophysis cells by promoting the expression of *Fshb* [28]. Long ncRNAs (LncRNAs) are a type of RNA that can participate in various biological processes by affecting gene transcription, protein activity, and epigenetic modification [29]. Our previous study showed that lncRNAs differ between immature and mature rat adenohypophyses [30]. In addition, we found that lncRNA-m433s1 can target miR-433 binding to regulate FSH secretion [31] and lncRNA-m18as1 can bind competitively to miR-18a-5p to regulate follicle-stimulating hormone secretion through the Smad2/3 pathway [32]. However, whether GnRH treatment can change the expression of lncRNAs in the rat adenohypophysis and the downstream regulatory mechanisms has not yet been reported. Additionally, the functional resolution of lncRNAs lacks relevant validation, and in addition, there is a lack of data supporting the regulatory network regarding the integrated regulatory relationships of lncRNAs, miRNAs, and mRNAs before and after GnRH treatment. In addition, it remains unclear whether noncoding RNAs are involved in the activation of Ca^2+^/CAMK II by GnRH to affect FSH synthesis and secretion.

In this study, we performed RNA sequencing of the rat adenohypophysis before and after GnRH treatment and detected the differential expression of mRNA, miRNA, and lncRNA in the adenohypophysis. Meanwhile, using bioinformatics software, we predicted the ceRNA regulatory network and experimentally elucidated the molecular mechanism of lncRNA regulation of FSH secretion. Our results provide data supporting the molecular mechanisms in the regulation of pituitary function by GnRH and complement the endocrine mechanisms of GnRH regulation of pituitary FSH secretion at the epigenetic level.

## 2. Materials and Methods

### 2.1. Ethics Statement

The experiments were carried out in strict accordance with the Guidelines for the Care and Use of Laboratory Animals of Jilin University. The animal experiments in this study were carried out in the Animal Experiment Center of Jilin University, and the experimental protocol of this study was approved by the Institutional Animal Care and Use Committee of Jilin University with the license number SY201809010.

### 2.2. Animals and GnRH Treatment

The animals used in the experiments were 8-week-old male Sprague Dawley (SD) rats, provided by the laboratory Animal Center of Jilin University. The grouping strategy used for this study has been described previously [22]. Briefly, 12 SD rats were equally divided into four groups: two control groups and two experimental groups. The rats in the experimental group were injected intra-abdominally twice with 0.2 mL of 1 µg/mL GnRH solution, with an interval of 2 h between injections. The rats in the control group were injected with the same dose of normal saline. All SD rats were euthanized with carbon dioxide ten minutes after the second injection, and the isolated adenohypophyses were placed in tubes containing TRIzol and stored at −80 °C until RNA extraction.

### 2.3. RNA Extraction

The rat adenohypophyses were removed from the −80 °C freezer, and total RNA was extracted by the TRIzol method. Then, a NanoDrop 2000 spectrophotometer (Thermo Fisher Scientific, Wilmington, DE, USA) was used to measure the concentration and purity of the total RNA in the rat adenohypophysis tissue. Agarose gel electrophoresis was performed to ensure that the total RNA from the rat adenohypophysis tissue was not contaminated. Finally, we used an RNA Nano 6000 Assay Kit and an Agilent Bioanalyzer 2100 system (Agilent Technologies, CA, USA) to detect whether the RNA was intact.

### 2.4. Library Construction

We set the GnRH-treated rats as the experimental group (L1, L2, S1, S2) and the saline-treated rats as the control group (L3, L4, S3, S4). Three adenohypophyses were used to construct two types of anterior adenohypophysis pools for each sample.

To construct cDNA libraries of lncRNAs and mRNAs, the ribosomal RNA (rRNA) in samples was removed using a Ribo-Zero kit (Epicenter, Madison, WI, USA), and then random fragmentation of the rRNA-depleted RNA was performed with fragmentation buffer. After the rRNA in the sample was removed, first-strand cDNA was synthesized using reverse transcriptase and random primers using the treated RNA as a template. Then, RNase H and DNA polymerase were added to the system to synthesize the second-strand cDNA. The DNA fragments were ligated to NEBNext Adapters after 3′ end adenylation. AMPure XP Beads (Beckman Coulter, Beverly, MA, USA) were used to purify the library fragments to select insertion fragments of 150~200 bp. Then, 3 µL of USER Enzyme (Beckman Coulter, Beverly, MA, USA) was used to process the cDNA to complete the ligation at 37 °C for 15 min. Finally, PCR was used to obtain the cDNA library.

A miRNA small RNA library was constructed with an NEBNext^®^ Small RNA Library Prep Set for Illumina (NEB, USA). Briefly, the 3′SR Adapter was first ligated; then, to prevent the formation of adapter dimers, we used SR-RT primers to bind the remaining 3′SR Adapter. Finally, the 5′ adapter was connected, and the first-strand cDNA was synthesized through reverse transcription. PCR was used to amplify the first-strand cDNA, and fragment screening was completed via polyacrylamide gel electrophoresis (PAGE).

BioMarker Technologies (Beijing, China) used the Illumina HiSeq X Ten platform to sequence our constructed libraries.

### 2.5. Sequencing, Clustering, Quality Control, and Inter-Sample Correlation Comparison

For lncRNAs and mRNAs, a TruSeq PE Cluster Kit v3-cBot-HS (Illumina, San Diego, CA, USA) was used to cluster the index-coding samples on the cBot Cluster Generation System. After clustering, the library preparation was sequenced on the Illumina HiSeq platform, and paired-end reads were generated.

MiRNAs were clustered on index-encoded samples using the TruSeq PE Cluster Kit v4-cBot-HS (Illumina) on the cBot Cluster Generation System. After cluster generation, library preparations were sequenced on the Illumina platform, and single-end reads were generated.

Quality control of the sequencing results was conducted. We first processed the raw data (raw reads) with a Perl script. In this process, clean reads are obtained by deleting low-quality data from the raw reads. At the same time, we calculated the Q30, Q20, and GC content of the clean reads based on the sequencing results [33]. The downstream analyses of the sequencing results were all performed on high-quality clean reads. For miRNA, it was necessary to screen for clean reads by deleting sequences shorter than 15 nt or longer than 35 nt.

Pearson’s correlation coefficient (r) was calculated to evaluate the correlations of biological replicates. The closer the correlation coefficient is to 1, the higher the similarity of expression patterns between samples.

### 2.6. Identification of lncRNAs, mRNAs, and miRNAs

We identified lncRNAs, mRNAs, and miRNAs through several methods using the rat reference genome (Rnor_6.0) to complete the alignment and analysis of the sequencing results.

For lncRNAs and mRNAs, we used StringTie [34] for transcriptome assembly, followed by the gff compare program for annotation of the assembled transcripts. By combining several computational tools, such as CPC2, CNCI, Pfam, and CPAT [35,36,37,38], we sorted the ncRNA candidates from the unknown transcripts and selected transcripts with lengths greater than 200 nt and with more than two exons as lncRNAs.

For miRNA, Bowtie [39] software was used to align the clean reads with reads in four databases: Silva, GtRNAdb, Repbase, and Rfam. The unannotated reads containing miRNAs were obtained. Then, Bowtie software was used to compare the unannotated reads and the abovementioned rat reference genome to obtain information on the positions of the reads in the rat genome. After the alignment was complete, we aligned the reads with miRBase to identify known miRNAs and finally used Randfold software to predict new miRNAs from the sequences of unknown miRNAs.

### 2.7. Differential Expression Analysis

The expression levels of mRNAs and lncRNAs in the sequencing results were calculated based on the fragments per kilobase of transcript per million mapped reads (FPKM) index, and then the DESeq [40] R package was used to analyze the differential expression of genes between the experimental group and the control group. To prevent false-positive results, we used the Benjamini and Hochberg method to adjust the resulting *p* values. Our criteria for screening differentially expressed mRNAs/lncRNAs were a |log2(fold change, FC)| ≥ 1 and a *p* value ≤ 0.01.

For miRNAs, we used the transcripts per million (TPM) algorithm [41] for normalization, followed by the DESeq2 [42] R package to identify differentially expressed miRNAs between the experimental and control groups. Again, the resulting *p* values were adjusted using the Benjamini and Hochberg method. Our criteria for identifying differentially expressed miRNAs were a |log2(FC)| ≥ 0.5 and a *p* value ≤ 0.05.

### 2.8. Target Gene Prediction

For lncRNA target gene prediction, we defined adjacent genes within 100 kb of lncRNAs as lncRNA cis-target genes, and we defined genes complementary to lncRNA sequences as lncRNA trans-target genes.

To investigate whether lncRNAs can affect gene expression by binding to specific miRNAs, we used the miRanda (animal), RNAhybrid, and TargetScan databases to predict the miRNAs targeting lncRNAs and mRNAs. We used the lncRNA, mRNA, and miRNA base sequence files from our RNA-seq results as input. Finally, we selected the differentially expressed lncRNAs, mRNAs, and miRNAs to construct a ceRNA regulatory network.

### 2.9. Enrichment Analysis

The differentially expressed genes, lncRNA cis-target genes, lncRNA trans-target genes, and miRNA target genes in our samples were used for enrichment analysis. ClusterProfiler was used to determine the enriched terms in the biological process, molecular function, and cellular component categories. The enrichment analysis employed a hypergeometric test approach to find GO entries that were significantly enriched for the differentially expressed compared to the whole genomic background. GO cluster analysis enabled us to investigate the clusters of biological functions of the differentially expressed genes. The Kyoto Encyclopedia of Genes and Genomes (KEGG) database was used to further interpret the functions of genes by performing pathway annotation.

### 2.10. Cell Culture

Rat adenohypophysis cells were isolated and digested from adenohypophysis tissue that was obtained from 8-week-old male SD rats. D/F12 (DMEM/F12, HyClone, Logan, UT, USA), containing 10% fetal bovine serum (FBS, Gibco, New York, NY, USA), as well as 1% penicillin and streptomycin, was used to culture rat adenohypophysis cells. Experiments were performed in a sterile environment, and adenohypophysis cells were cultured in a 5% CO_2_ incubator at 37 °C. The detailed methods for rat primary cell culture were consistent with those described in previous studies [43].

### 2.11. Cell Transfection

Cells were inoculated into 12-well plates at a cell count of 3 × 10^5^ cells per well, and the cell density after inoculation was 70%. Cells were cultured in DMEM/F12 containing 10% fetal bovine serum (FBS) and 1% penicillin and streptomycin (Gibco) to reach 50% cell density. First, we added buffer, reagents, mimics, and inhibitors to the centrifuge tubes. Then, we briefly centrifuged the samples using a vortex shaker to ensure homogeneous mixing. Next, we transfected the mixture into a 12-well plate filled with adenohypophysis cell suspension. Finally, we placed the 12-well plates into a 5% CO_2_ incubator to provide sufficient time for the reaction. The siRNA, mimic, and inhibitor for the transfection process were obtained from Ribo (Guangzhou, China), and the transfection process was carried out strictly according to the protocol recommended by Ribo.

### 2.12. Real-Time Quantitative PCR Assay

We obtained cDNA by reverse transcription using a FastQuant RT Kit (with gDNase, Tiangen, Beijing, China). Next, we detected the mRNA expression of related genes by RT-qPCR using MonAmpTM ChemoHS qPCR Mix (Mona Bio, Wuhan, China). All primers were designed by the NCBI (Table S19). The reference gene for mRNA was GHPDH. The reference gene for miRNA was U6.

### 2.13. Dual Luciferase Reporter Gene Assay

The dual luciferase reporter used the pmiR-RB-REPORT™ dual luciferase reporter vector. The reporter fluorophore of the vector was the sea kidney luciferase gene (hRluc). The internal reference was the firefly luciferase gene (hluc) and clones the target gene downstream of the hRluc gene. Since the predicted obtained miRNAs may target the target gene, miRNA mimics were co-transfected with the constructed vectors in cells, and the interaction between miRNAs and target genes was verified by the downregulation of the relative fluorescence values of reporter genes. The plasmids used in the transfection process were obtained from Ribo (Guangzhou, China), and the transfection process was performed strictly according to the protocol recommended by Ribo.

### 2.14. Western Blot Analysis

After we lysed the cells, the concentration of protein samples was first determined. Next, after separating the proteins by PAGE gel electrophoresis, the proteins were transferred to PVDF membranes (Millipore, Carlsbad, CA, USA), and the PVDF membranes were blocked at room temperature for 15 min. Then, the PVDF membranes were incubated with primary antibodies. The next day, the membrane was washed three times using TBST and incubated with secondary antibodies for 1 h. After washing again, the membrane was developed on a Tanon 5200 Multifunctional System (Tanon, Shanghai, China). The antibodies used in this experiment were anti-GAPDH (1:1000, Cell Signaling Technology, Shanghai, China, 2118S), anti-CAMK2D (1:1000, Abcam, Cambridge, MA, USA, ab181052), and anti-rabbit IgG (H+L)-HRP (1:3000; Cell Signaling Technology, Shanghai, China, 7074S). All details are provided in Support File S1.

### 2.15. ELISA

After collecting the supernatant, we used the Rat FSH ELISA kit (EnzymeLink Biotechnology Co., Ltd., Shanghai, China) to detect changes in the levels of FSH. All steps were performed strictly according to the manufacturer’s protocol. The standard curve was drawn according to the concentrations of the standards provided by the kit, and the concentration of each sample was calculated.

### 2.16. Statistical Analysis

The data are presented as the mean from three independent experiments ± standard deviation of the mean. Significant differences between groups were determined using SPSS 22.0 software and one-way ANOVA, and *p* < 0.05 was considered to indicate statistical significance.

## 3. Results

### 3.1. Expression Analysis of mRNAs, lncRNAs, and miRNAs in the Rat Adenohypophysis

We performed quality control on the original sequencing reads to obtain high-quality clean reads. In this study, a total of 4 libraries with a total of 71.51 Gb of clean data were analyzed for lncRNAs (Table S1). For miRNAs, a total of 78.83 M clean reads were obtained from the sequencing results (Table S2). Afterward, we aligned the clean reads of each sample with the rat reference genome (Rattus_norvegicus.Rnor6.0) to analyze the sequence feature information of each sample. We compared the lncRNA sequencing results from rats with the rat reference genome and found that at least 94.77% of the reads mapped to the rat genome (Table S3). For miRNAs, statistical analysis revealed that at least 67.44% of the unannotated reads were miRNAs (Table S4). We used the Pearson correlation coefficient r to evaluate the biological replicate correlations of the samples, and the r2 statistics of the lncRNA, mRNA, and miRNA samples are shown in Supporting Tables S5–S7.

**Figure 1:**
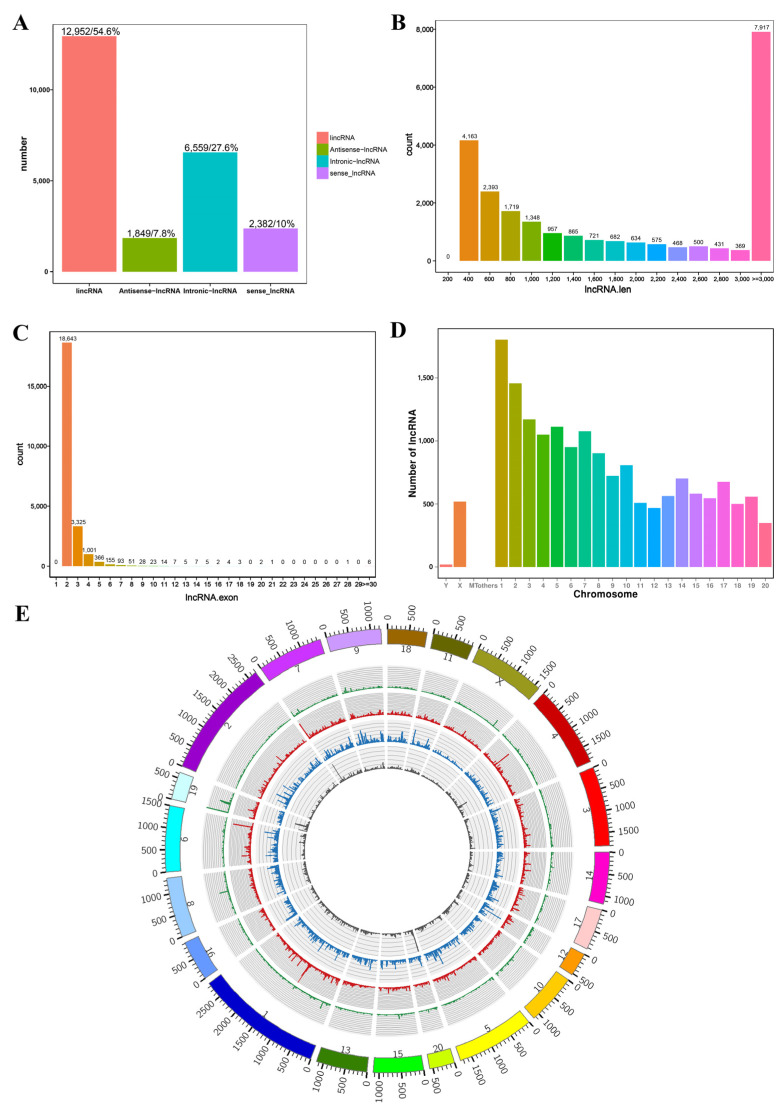
Results of lncRNA sequencing in the adenohypophysis of rats after GnRH treatment. (**A**) LncRNA types and numbers. (**B**) Statistical analysis of LncRNA lengths. (**C**) Statistical analysis of LncRNA exon number. (**D**) Locational distribution of lncRNAs on rat chromosomes. (**E**) Circos plot containing lncRNA data.

We performed RNA-seq to examine the expression of lncRNAs, mRNAs, and miRNAs in the adenohypophyses of rats before and after GnRH treatment. We found that a total of 23,742 lncRNAs were expressed in the rat adenohypophysis, including 1849 antisense lncRNAs (8%), 6559 intronic lncRNAs (28%), 12,952 long intergenic ncRNAs (lincRNAs) (55%), and 2382 sense lncRNAs (10%) (Figure 1A). The lncRNA lengths were distributed between 400 and 3000+ nt, but most lncRNAs were distributed in the 3000+ nt range, while the others were mainly distributed in the 400–1000 nt range (Figure 1B). The lncRNAs were mainly distributed on chromosomes 1–8 (Figure 1C). We also found that these lncRNAs were expressed in all chromosomes of the rat genome but were mainly distributed on chromosomes 1–10 (Figure 1D). Finally, we mapped the sequenced lncRNAs into the genome and drew a Circos map (Figure 1E).

**Figure 2:**
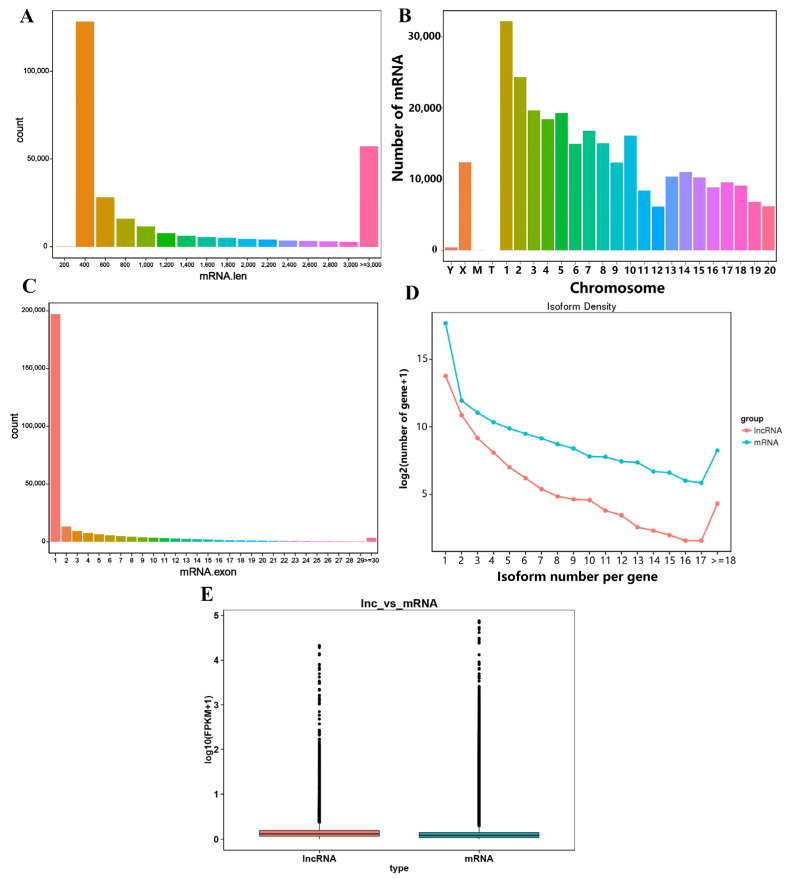
Results of mRNA sequencing in the adenohypophysis of rats after GnRH treatment. (**A**) Statistical analysis of mRNA length. (**B**) Location distribution of mRNA on rat chromosomes. (**C**) Statistical analysis of mRNA exon numbers. (**D**) mRNA/lncRNA alternatively spliced isoform levels. (**E**) Comparison of mRNA/lncRNA expression levels.

For mRNAs, we found that a total of 27,367 genes were expressed in the rat adenohypophysis. Then, based on the alignment results we discovered a total of 9638 new genes, of which 6084 were functionally annotated (Table S8). The mRNA lengths were distributed between 200 and 3000+ nt, but most lncRNAs were distributed in the 400–800 nt range, while the others were mainly distributed in the 3000+ nt range (Figure 2A). These mRNAs were distributed on all chromosomes in the rat genome, but were most highly expressed on chromosome 1 (Figure 2B). Most mRNAs contained exons clustered between 1 and 2 (Figure 2C). Upon comparing the sequenced lncRNAs and mRNAs, we found that the number of alternatively spliced isoforms of mRNAs was higher than the overall number of lncRNAs (Figure 2D). The boxplots clearly show that the expression levels of mRNA were slightly lower than those of lncRNA transcripts (Figure 2E).

**Figure 3:**
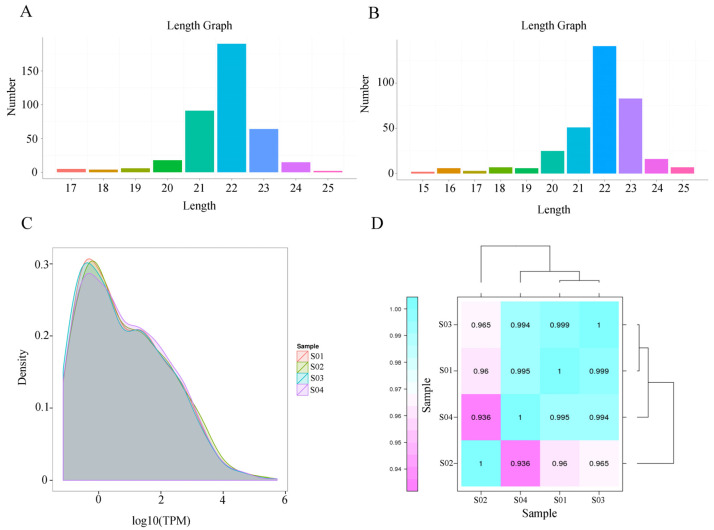
Results of miRNA sequencing in the adenohypophysis of rats after GnRH treatment. (**A**,**B**) Statistical analysis of miRNA length. (**C**) Overall distribution of miRNA expression. The curves of different colors in the figure represent different samples, the horizontal coordinates of the points on the curves indicate the logarithmic values of the TPM of the corresponding samples, and the vertical coordinates of the points indicate the probability density. (**D**) Sample correlation relationship chart. Different colors in the graph represent different correlation coefficient values. The horizontal and vertical coordinates represent different samples.

A total of 742 miRNAs were also detected in the RNA-seq results, including 395 known mRNAs and 347 newly predicted miRNAs (Table S9). The identified known miRNAs and new miRNAs as well as the overall miRNA length were mainly concentrated in the range of 20 nt to 24 nt, with 22 nt predominant (Figure 3A,B). Among the detected miRNAs, a small number of miRNAs were expressed at high levels, and the expression of most of the miRNAs was still relatively low (Figure 3C). In addition, the correlation coefficients between all four groups of samples were above 0.93, which implies similar expression trends and high replication correlations between the samples (Figure 3D).

### 3.2. Analysis of Differential Gene Expression before and after GnRH Treatment

**Figure 4:**
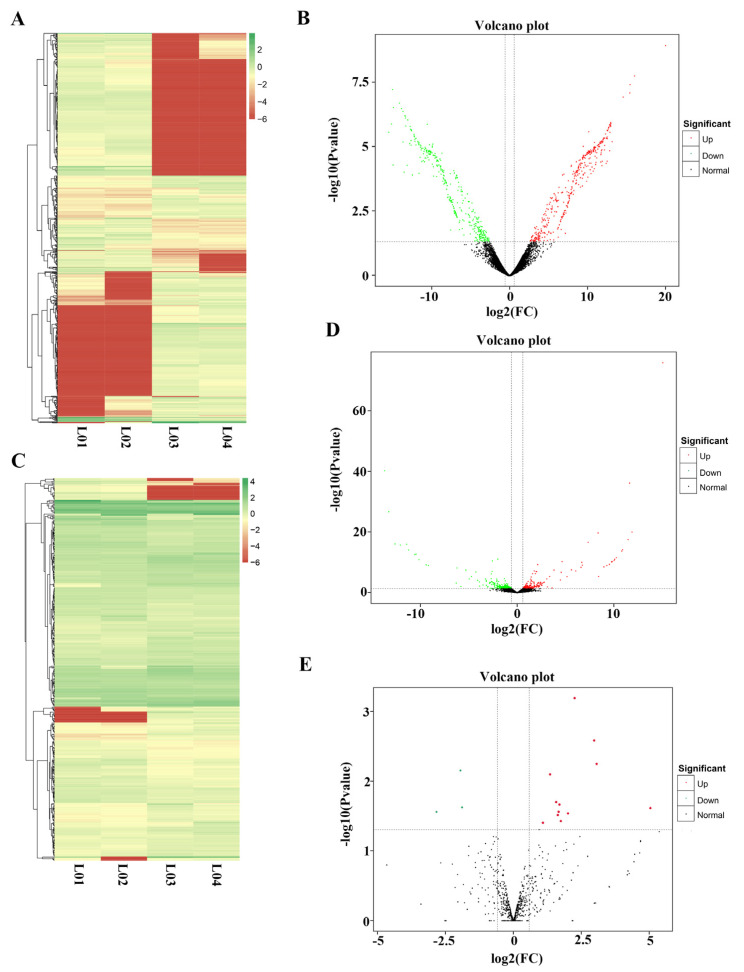
Analysis of differential gene expression before and after GnRH treatment. (**A**) Differential expression analysis of four groups of lncRNAs. (**B**) LncRNA expression volcano plot. (**C**) Differential expression analysis of four groups of mRNAs. (**D**) mRNA expression volcano plot. (**E**) miRNA expression volcano plot.

After statistical analysis of the expression of all lncRNAs, mRNAs, and miRNAs in the samples, excluding RNAs that were expressed individually in only a single sample, DESeq was used to detect differential expressions of RNAs after GnRH treatment. For detection of significantly differentially expressed lncRNAs, a *p* value ≤ 0.01 and |log2(FC)| ≥ 1 were used as the screening criteria (Figure 4A). Ultimately, we obtained 704 significantly differentially expressed lncRNAs, of which 366 were significantly upregulated after GnRH treatment and 338 were significantly downregulated after GnRH treatment. (Figure 4B and Table S10).

We used a *p* value ≤ 0.01 and a |log2(FC)| ≥ 1 as screening criteria for significantly differentially expressed mRNAs (Figure 4C). Ultimately, 385 differentially expressed mRNAs were obtained, of which 180 mRNAs were significantly upregulated after GnRH treatment and 205 mRNAs were significantly downregulated after GnRH treatment (Figure 4D and Table S11).

Likewise, we used DESeq2 to detect differentially expressed miRNAs before and after GnRH treatment. Ultimately, 20 differentially expressed miRNAs were obtained, of which 13 miRNAs were significantly upregulated after GnRH treatment and 7 miRNAs were significantly downregulated after GnRH treatment (Figure 4E and Table S12).

### 3.3. Interactions among lncRNAs, mRNAs, and miRNAs

**Figure 5:**
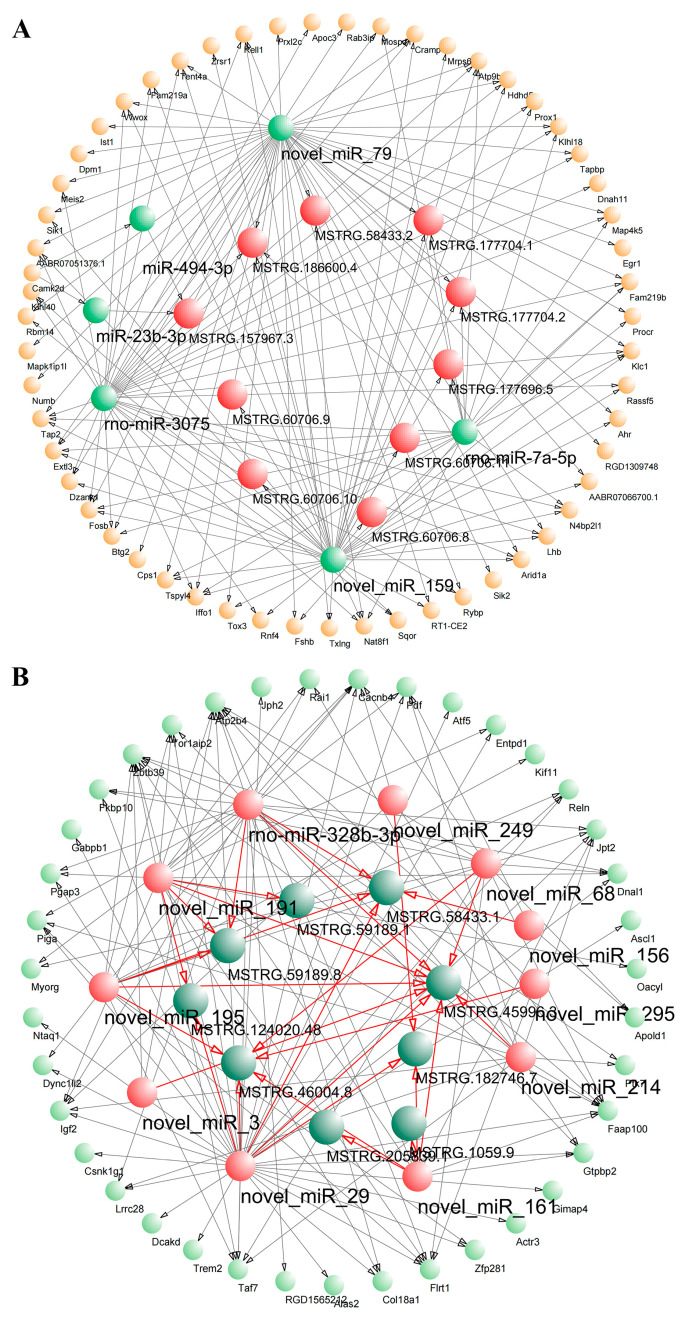
Interactions among lncRNAs, mRNAs, and miRNAs. (**A**) GnRH-promoting ceRNA regulatory network in the rat adenohypophysis. (**B**) GnRH-inhibitory ceRNA regulatory network in the rat adenohypophysis.

Since mRNAs and lncRNAs contain multiple miRNA response elements (MREs), software can be used to predict miRNAs that may target lncRNAs and mRNAs. We performed targeting relationship prediction for differentially expressed lncRNAs, mRNAs, and miRNAs after GnRH treatment. The prediction results showed that 366 upregulated lncRNAs, 7 downregulated miRNAs, and 190 upregulated mRNAs constituted a GnRH-promoted ceRNA regulatory network (Table S13); in addition, 338 downregulated lncRNAs, 13 upregulated miRNAs, and 203 downregulated mRNAs constituted a GnRH-repressive ceRNA regulatory network (Table S14). Finally, Cytoscape software was used to select the ten lncRNAs with the highest expression levels to generate a circular lncRNA–miRNA–mRNA regulatory network map (Figure 5A,B).

The GO analysis results for the GnRH-promoted ceRNA regulatory network showed that the differentially expressed genes were mainly enriched with 51 GO terms, including cellular processes, intracellular anatomical structures, and protein bindings (Table S15). KEGG showed that the ceRNA target genes were mainly enriched in the MAPK signaling pathway, the calcium signaling pathway, the cAMP signaling pathway, and other pathways (Table S16). The GO analysis results for the GnRH-mediated inhibition of the ceRNA regulatory network showed that the differentially expressed genes were mainly enriched with 73 GO terms, including intracellular organelles, bindings, and metabolic processes (Table S17). KEGG showed that the ceRNA target genes were mainly enriched in the GnRH signaling pathway, antigen processing, and presentation (Table S18).

### 3.4. GnRH Promotes the Target Binding of lncRNA-m23b to miR-23b-3p

**Figure 6:**
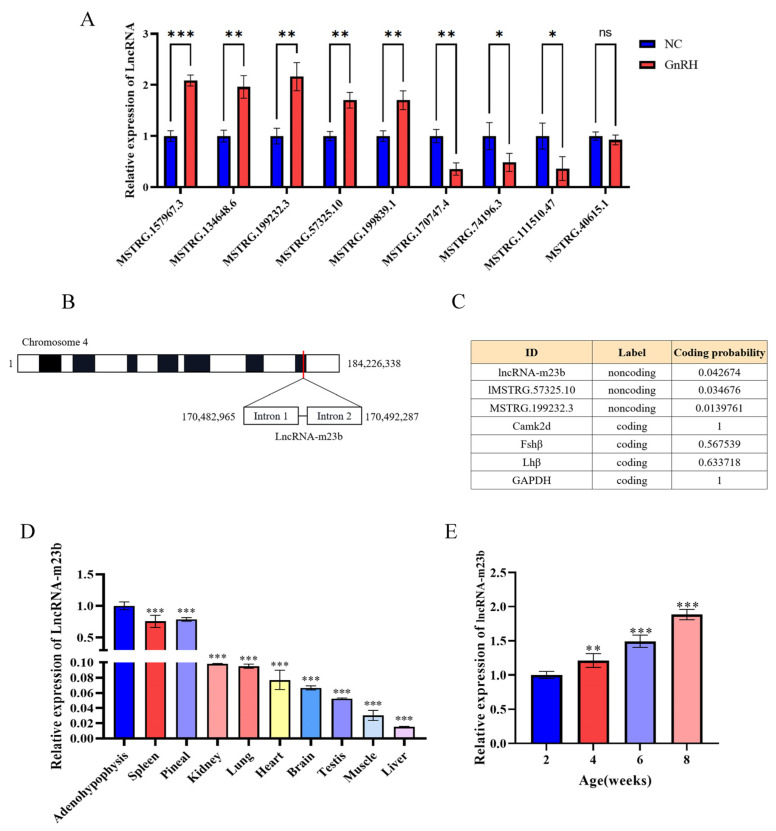
Identification of LncRNA-m23b. (**A**) Detection of differentially expressed lncRNAs by RT-qPCR. (**B**) LncRNA-m23b chromosomal information. (**C**) Prediction of coding potential by CPC 2.0. (**D**) Expression of lncRNA-m23b in different tissues of male rats, lncRNA-m23b expression in the adenohypophysis was used as a control. (**E**) Expression of lncRNA-m23b in the adenohypophysis of rats at different developmental stages. ns, *p* > 0.05; *, *p* < 0.05; **, *p* < 0.01; ***, *p* < 0.001.

Based on the above results, we selected nine lncRNAs as candidates for this study. RT-qPCR results showed that the expression of MSTRG.157967.3, MSTRG.134648.6, MSTRG.199232.3, MSTRG.57325.10, and MSTRG.199839.1 was significantly increased after GnRH treatment (Figure 6A). Based on the ceRNA regulatory network and validation results, lncRNA-MSTRG.157967.3 was finally identified as our candidate for study and renamed lncRNA-m23b. lncRNA-m23b is an intergenic lncRNA on chromosome 4, consisting of two introns (Figure 6B). We predicted its coding ability using the Coding Potential Calculator (CPC) 2.0 (http://cpc2.gao-lab.org, accessed on 3 January 2022), and the prediction showed that lncRNA-m23b has no coding ability (Figure 6C). We further investigated the expression of lncRNAs in different tissues of male rats. The results showed that lncRNA expression was the highest in the adenohypophysis gland, followed by the pineal gland, spleen, and kidneys. The expression was lower in other tissues (Figure 6D). We next identified the expression of lncRNA-m23b in the adenohypophysis of rats at different ages, and the results showed that the expression of lncRNA-m23b gradually increased with age (Figure 6E).

**Figure 7:**
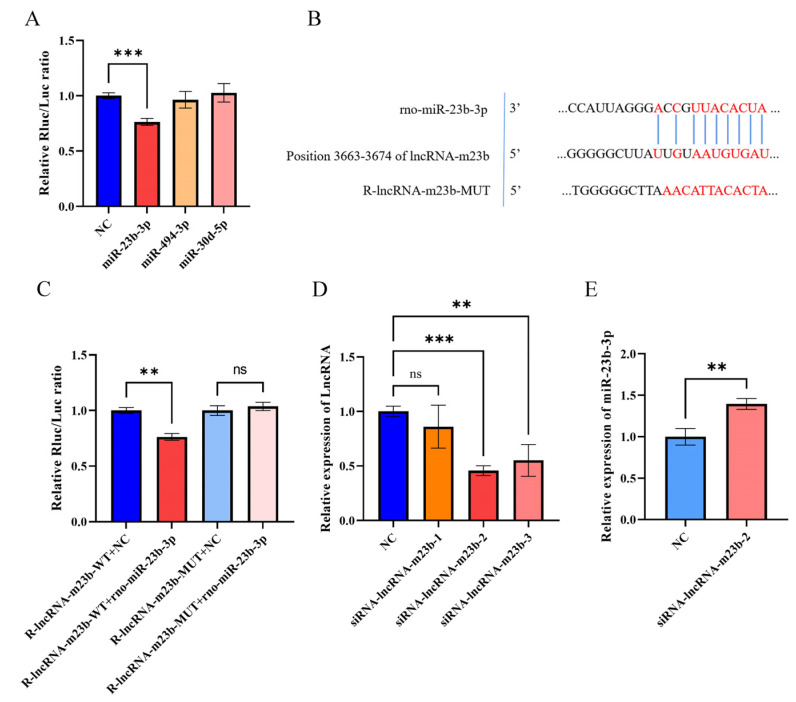
(**A**) Identification of miRNAs that might directly target lncRNA-m23b. (**B**) TargetScan’s anticipated miR-23b-3p base complementary pairing sequence with lncRNA-m23b is highlighted in red. (**C**) Changes in relative luciferase activity after the transfection of plasmids with NC/mimics. (**D**) Screening of siRNAs. (**E**) RT-qPCR after knockdown of lncRNA-m23b. ns, *p* > 0.05; **, *p* < 0.01; ***, *p* < 0.001.

To investigate the function of lncRNAs and how they function in cells, we used TargetScan, miRanda, and RNAhybrid to predict miRNAs that may have a targeting relationship with lncRNA-m23b. To determine the relationship between lncRNA-m23b and miRNAs, we performed dual luciferase reporter gene experiments, which showed that co-transfection of pmiR-lncRNA-m23b-WT with rno-miR-23b-3p resulted in a significant decrease of luciferase activity (Figure 7A). Then, based on the base complementation region information predicted by the TargetScan (Figure 7B), we mutated the targeted complementary sequences to construct the reporter gene mutant plasmid pmiR-lncRNA-m23b-MUT. After co-transfection, the relative luciferase activity of the mutant reporter vector was significantly restored compared to that of the wild-type reporter vector (Figure 7C). We next performed siRNA transfection of lncRNA-m23b in rat adenoidal cells, and the results showed that siRNA-lncRNA-m23b-2 had the best transfection effect after siRNA transfection (Figure 7D), so we chose siRNA-lncRNA-m23b-2 for subsequent transfection experiments. lncRNA-m23b knockdown was followed by a significant increase in the miR-23b-3p expression (Figure 7E).

### 3.5. LncRNA-m23b Is Involved in the Regulation of the FSH Synthesis and Secretion by Targeting the Camk2d Expression through Competitive Binding with miR-23b-3p

**Figure 8:**
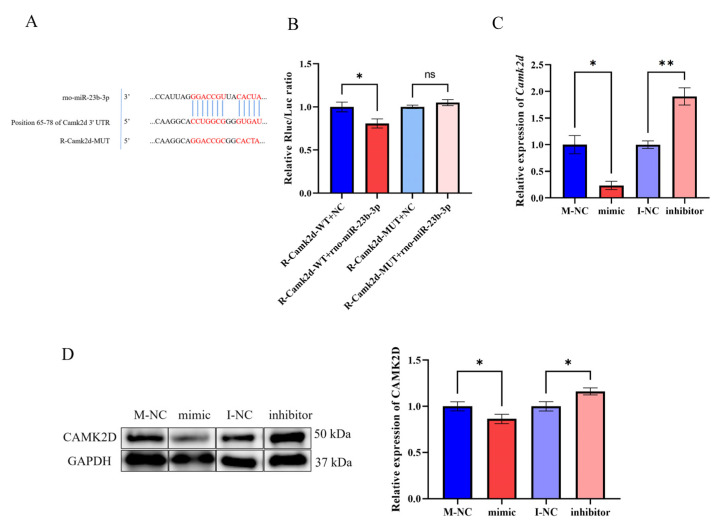
MiR-23b-3p targets Camk2d-3′UTR and regulates its gene expression. (**A**) TargetScan’s anticipated miR-23b-3p base complementary pairing sequence with lncRNA-m23b is highlighted in red. (**B**) Changes in relative luciferase activity after the transfection of plasmids with NC/mimic. (**C**,**D**) *Camk2d* mRNA and CAMK2D protein changes after miR-23b-3p mimic/inhibitor transfection. ns, *p* > 0.05; *, *p* < 0.05; **, *p* < 0.01.

We used TargetScan to predict the mRNAs that might target miR-23b-3p and found that the binding sites between miR-23b-3p and *Camk2d* were similar to those between miR-23b-3p and lncRNA-m23b (Figure 8A). Next, we co-transfected the constructed pmiR-Camk2d-3′UTR-WT reporter plasmid with the miR-23b-3p mimic and the negative control (NC), and found that transfection of the miR-23b-3p mimic resulted in a significant decrease of luciferase activity (Figure 8B). We mutated the targeted complementary sequence to construct a reporter gene mutant plasmid, pmiR-Camk2d-3′UTR-MUT, and the relative luciferase activity was significantly restored after co-transfection (Figure 8B). Next, we next performed miR-23b-3p mimic/inhibitor transfection in rat adenoidal cells. RT-qPCR results showed that overexpression of miR-23b-3p significantly downregulated *Camk2d* mRNA expression and inhibition of miR-23b-3p upregulated *Camk2d* mRNA expression (Figure 8C), while Western blotting results showed that miR-23b-3p overexpression resulted in a significant decrease in CAMK2D protein expression level, and inhibition of miR-23b-3p expression resulted in a significant increase in CAMK2D protein expression level (Figure 8D).

**Figure 9:**
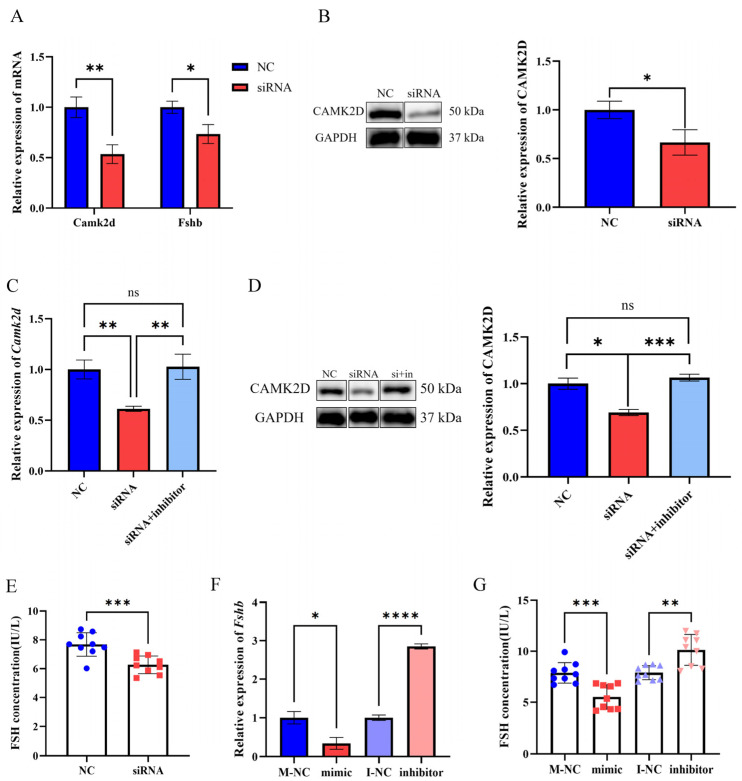
LncRNA-m23b is involved in the regulation of the FSH synthesis and secretion by targeting *Camk2d* expression through competitive binding with miR-23b-3p. (**A**) mRNA changes after lncRNA-m23b knockdown. (**B**) The CAMK2D protein changes after lncRNA-m23b knockdown. (**C**,**D**) *Camk2d* mRNA and CAMK2D protein changes after co-transfection of siRNA-lncRNA-m23b-2 with miR-23b-3p inhibitor. (**E**) FSH secretion changes after lncRNA-m23b knockdown. (**F**,**G**) *Fshb* mRNA and FSH secretion changes after miR-23b-3p mimic/inhibitor transfection. ns, *p* > 0.05; *, *p* < 0.05; **, *p* < 0.01; ***, *p* < 0.001; ****, *p* < 0.0001.

In addition, we found that *Camk2d* mRNA expression was significantly decreased after the lncRNA-m23b knockdown (Figure 9A). Western blotting results showed that CAMK2D protein expression was significantly decreased after the lncRNA-m23b knockdown (Figure 9B). We next performed rescue experiments to co-transfect siRNA-lncRNA-m23b-2 with a miR-23b-3p inhibitor. RT-qPCR results showed that inhibition of miR-23b-3p alleviated the inhibitory effect of lncRNA-m23b knockdown on *Camk2d* mRNA (Figure 9C). Western blotting results showed that inhibition of miR-23b-3p attenuated the inhibitory effect of lncRNA-m23b knockdown on the CAMK2D protein (Figure 9D). These results demonstrate that lncRNA-m23b regulates *Camk2d* expression through competitive binding of miR-23b-3p. In addition, we found that knockdown of lncRNA-m23b resulted in a significant decrease in FSH expression (Figure 9A). The ELISA results showed that FSH secretion was significantly decreased after lncRNA-m23b knockdown (Figure 9E). The overexpression of the miR-23b-3p mimic significantly downregulated *Fshb* mRNA expression, and inhibition of miR-23b-3p upregulated *Fshb* mRNA expression (Figure 9F). ELISA results showed that miR-23b-3p overexpression resulted in a significant decrease of FSH secretion, and inhibition of miR-23b-3p expression resulted in a significant increase of FSH secretion (Figure 9G).

## 4. Discussion

GnRH is involved in the regulation of the entire hypothalamic–pituitary–gonadal axis and plays an important role in reproductive regulation [44]. Our previous research revealed significant differences in methylation levels in the adenohypophyses of rats after GnRH treatment [24]. In addition, 14 circRNAs were found to be significantly differentially expressed in the rat adenohypophysis [22]. However, the overall regulatory effects of GnRH on the expression of other ncRNAs and mRNAs in the rat adenohypophysis have not been reported. Therefore, in this study, RNA-seq was used to detect the expression changes of lncRNAs, mRNAs, and miRNAs in the adenohypophyses of rats before and after GnRH treatment.

Regarding the differentially expressed mRNAs, RNA-seq showed that genes including *Egr1*, *Map4k5*, *Fosb*, *Myh11,* and *Pkm* were significantly upregulated in the adenohypophyses of rats after GnRH treatment. The regulation of some differentially expressed mRNAs by GnRH has been verified in other studies. For example, different frequencies of GnRH pulses can upregulate the expression of *Egr1* through MAPK 8/9 and MAPK1/3, and early growth factor (EGR1) is crucial for *Lhb* transcription [45]. During GnRH-mediated regulation of gonadotropin expression, MAPK affects gonadotropin transcription and plays an important role in intracellular signal transduction [46]. Other upregulated genes also play important roles in reproductive regulation after GnRH treatment [47,48,49]. Previous research has shown that GnRH treatment with different pulse frequencies can significantly increase the levels of phospho-CAMK2, which can mediate intracellular calcium signaling pathways [16,50]. In addition, GnRH can regulate the expression of *Fshb* by activating the MAPK signaling pathway [16].

Regarding the differentially expressed miRNAs, the sequencing results showed that rno-miR-3075 and rno-miR-7a-5p were significantly downregulated after GnRH treatment. Moreover, there is a targeting relationship between miR-3075 and *Fosb* in rats [51]. Another study has demonstrated that GnRH treatment can significantly upregulate the expression of *Fosb* [52]. In addition, rno-miR-7a-5p has been shown to regulate the expression of *Fshb* in the rat adenohypophysis [53]. The above findings support the accuracy of our RNA-seq results. However, other studies have found that various miRNAs, including rno-miR-186-5p, rno-miR-433, and rno-miR-488, can regulate the synthesis of *Fshb* [23,31,54], and our sequencing results did not indicate significant differential expression of these miRNAs, possibly because the differences in the expression of these miRNAs did not meet our threshold for significance or because GnRH did not have a regulatory effect on these miRNAs.

LncRNAs are ncRNAs, and increasing evidence indicates that lncRNAs are involved in the mammalian reproductive regulation process [55,56,57]. In general, lncRNAs can act as molecular sponges to competitively bind miRNAs to regulate gene expression; this is referred to as the intracellular ceRNA regulatory mechanism [58]. An increasing number of studies have shown that ceRNA regulatory networks play important roles in the reproductive process. For example, the lncRNA PATR can competitively bind to miR-101-3p to regulate the expression of ZEB1, thereby promoting epithelial–mesenchymal transition (EMT) [59]. In addition, studies have found that in the rat adenohypophysis, lncRNA-m433s1 can act as a molecular sponge for miR-433 and participate in reproductive processes by regulating the expression of *Fshb* [31]. The results of our enrichment analysis indicated that the lncRNAs and mRNAs that were differentially expressed after GnRH treatment may play important roles in reproductive regulation. Therefore, we screened out the differentially expressed mRNAs, lncRNAs, and miRNAs and constructed a GnRH-promoted ceRNA regulatory network and a GnRH-inhibited ceRNA regulatory network for rat glands based on their regulatory relationships. We performed GO and KEGG enrichment analyses on the genes in the two ceRNA regulatory networks. KEGG enrichment analysis revealed that the target genes in the two ceRNA regulatory networks were enriched in GnRH targeting-related pathways. Together, the above enrichment results indicate that the differentially expressed lncRNAs may be regulated by GnRH and may participate in various biological processes in the rat adenohypophysis through different pathways.

Generally, lncRNAs are able to regulate gene expression by competitively binding to miRNAs in the cytoplasm [60]. Our results showed that GnRH treatment was able to induce an increase in lncRNA-m23b expression levels after GnRH treatment, while our study established the function of lncRNA-m23b as a miRNA sponge. First, the dual-luciferase reporter assay showed that lncRNA-m23b has a targeting relationship with miR-23b-3p, and the 3′UTR of *Camk2d* mRNA also has a targeting binding site for miR-23b-3p. Second, knockdown of lncRNA-m23b leads to an upregulation of miR-23b-3p expression and a repression of *Camk2d* mRNA expression. Moreover, overexpression of miR-23b-3p leads to repression of *Camk2d* mRNA expression, and conversely, inhibition of miR-23b-3p leads to elevated expression of *Camk2d* mRNA. To demonstrate that the inhibition of *Camk2d* mRNA expression was caused by the increase in miR-23b-3p, we performed co-transfection experiments of siRNA-lncRNA-m23b with a miR-23b-3p inhibitor in cells. Expression of *Camk2d* was significantly recovered by inhibiting the increase in miR-23b-3p levels that would normally occur in the absence of lncRNA-m23b. Therefore, based on the ceRNA hypothesis, we identified a miR-23b-3p-mediated ceRNA mechanism between lncRNA-m23b and *Camk2d*.

Calcium/calmodulin-dependent kinase II (Ca^2+^/CAMK II) is an important mediator of calcium signaling in various cell types and is also able to regulate gene expression and hormone secretion [61,62]. It has been reported that GnRH can affect *Fshb* gene expression and thus enhance FSH secretion by activating Ca^2+^/CAMK II [19]. In the present study, we found that *Camk2d* is a target gene of miR-23b-3p and that lncRNA-m23b regulates *Camk2d* by competitively binding miR-23b-3p. We also examined the secretion of *Fshb* mRNA as well as FSH hormone after transfection. The results showed that the expression trend of *Fshb* was consistent with the expression trend of *Camk2d*. Therefore, we hypothesized that GnRH treatment could cause an increase in the expression level of lncRNA-m23b, which could compete with miR-23b-3p to increase the expression of *Camk2d* mRNA and ultimately promote the synthesis and secretion of FSH. Due to the many pathways involved in the regulation of FSH synthesis and secretion by Ca^2+^/CAMK II, we did not investigate them in depth in this paper. In future studies, we will further reveal the mechanism of GnRH regulation of FSH synthesis and secretion.

Due to the complexity of the female physiological cycle and the vast differences in the regulation between gonadotropin-releasing hormone and gonadotropins in females at different physiological stages, the most fundamental issue of our research is the synthesis of gonadotropins. This adds to the difficulties of our study. At the same time, it is possible that the results we obtained are only appropriate for a particular physiological period. However, there are fewer factors involved in male than in female animals. Therefore, we chose only male rats for our study to obtain a more generalized result and thus enrich the molecular mechanism of GnRH regulation of FSH secretion. As our research into the mechanisms of FSH synthesis progresses, we will consider exploring more detailed molecular mechanisms of FSH synthesis in animals at different physiological stages in the future.

**Figure 10:**
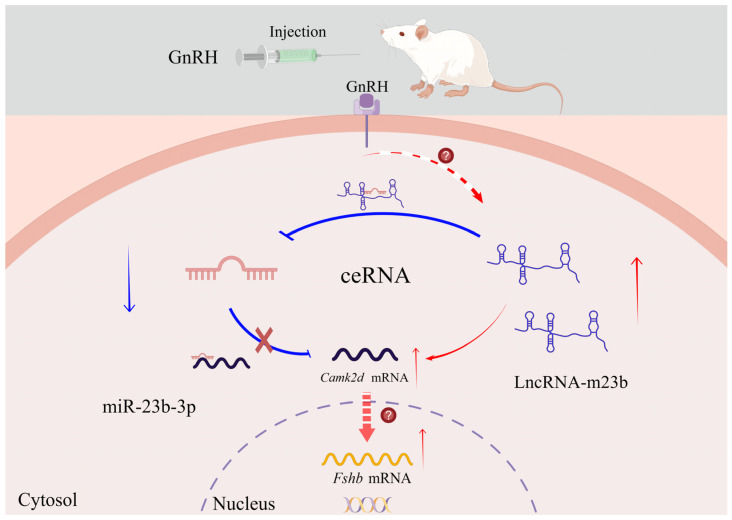
Schematic of the proposed lncRNA-m23b mechanism in rat anterior adenohypophysis cells. The blue arrows on the diagram represent inhibition, and the red arrows represent facilitation. This figure was drawn using Figdraw (https://www.figdraw.com/static/index.html#/, accessed on 2 March 2023). The unique authorization code is UOWUU765ca.

In conclusion, this is the first study to analyze the overall expression changes of mRNAs/lncRNAs/miRNAs in the adenohypophyses of rats after GnRH treatment. We identified mRNAs, lncRNAs, and miRNAs in the rat adenohypophysis before and after GnRH treatment and constructed a ceRNA regulatory network composed of the differentially expressed mRNAs, lncRNAs, and miRNAs in the rat adenohypophysis. This network may play an important regulatory role in GnRH-mediated regulation of the rat adenohypophysis and reproduction. In addition, we performed enrichment analysis on the differentially expressed mRNAs, lncRNA target genes, and ceRNA regulatory networks to analyze their potential roles in the rat adenohypophysis. We demonstrated for the first time that GnRH can affect FSH synthesis and secretion by regulating the overexpression of lncRNA-m23b and promoting the competitive binding of lncRNA-m23b to miR-23b-3p to regulate *Camk2d* (Figure 10). We hope that our findings will provide a reference for researchers who are interested in adeno-pituitary ncRNAs and aid in further exploration of the regulatory role of GnRH in the pituitary.

## 5. Conclusions

In conclusion, this study analyzed the overall lncRNA/mRNA/miRNA expression changes in the rat adenohypophysis after GnRH treatment. It was also confirmed that GnRH can affect the synthesis and secretion of FSH by overexpressing lncRNA-m23b and promoting the competitive binding of lncRNA-m23b to miR-23b-3p, which in turn regulates the overexpression of *Camk2d*. Our study provides a theoretical basis for resolving the regulatory role of GnRH in the adenohypophysis gland.

## Data Availability

All sequencing data from this study can be found in the supporting documentation.

## Supplementary Materials

The following supporting information can be downloaded at: https://www.mdpi.com/article/10.3390/genes14040846/s1, Support File S1: Western blot analysis. Support table: Table S1: Statistical table of LncRNA sequencing data evaluation, Table S2: Statistical table of miRNA sequencing data evaluation, Table S3: Statistical table of LncRNA Clean Data and rat genome comparison results, Table S4: Statistical table of miRNA Clean Data and rat genome comparison results, Table S5: LncRNA sample correlation coefficient, Table S6: mRNA sample correlation coefficient, Table S7: miRNA sample correlation coefficient, Table S8: Annotation of new genes, Table S9: miRNA expression, Table S10: lncRNAs with differential expression, Table S11: mRNAs with differential expression, Table S12: miRNAs with differential expression, Table S13: GnRH-promoting ceRNA regulatory network, Table S14: GnRH-inhibitory ceRNA regulatory network, Table S15: GO annotation of the GnRH-promoting ceRNA regulatory network, Table S16: KEGG annotation of GnRH-promoting ceRNA regulatory network, Table S17: GO annotation of GnRH-inhibitory ceRNA regulatory network, Table S18: KEGG annotation of GnRH-inhibitory ceRNA regulatory network, Table S19: All primers.
