# Supplementary material for: Sequencing of the Pituitary Transcriptome after GnRH Treatment Uncovers the Involvement of lncRNA-m23b/miR-23b-3p/CAMK2D in FSH Synthesis and Secretion
Source: Genes (Basel). 2023 Mar 31;14(4):846. doi: 10.3390/genes14040846 (PMC10137480; doi:10.3390/genes14040846)
Supplement: Supplementary file 1 [file genes-14-00846-s001.zip › Support File S1.pdf]

Support File 1

Western blot analysis

We lysed pituitary cells using RIPA lysis buffer (Yase, Shanghai, China) containing 1 mM phenylmethylsulfonyl fluoride (PMSF). We determined the concentration of protein samples using the BCA protein assay kit (Beyotime, Shanghai, China) according to the manufacturer's instructions. After protein separation by PAGE gel electrophoresis, proteins were transferred to polyvinylidene difluoride (PVDF) membranes (Millipore), and then the PVDF membranes were blocked at room temperature for 15 min with rapid closure solution (Yase), followed by incubation of the PVDF membranes in diluted specific primary antibody at 4°C overnight. The next day, PVDF membranes were washed 3 times (10 min each time) in TBST buffer and then incubated in secondary antibody at room temperature for 1 h. After washing, PVDF membranes were developed on a Tanon 5200 Multi System (Tanon, Shanghai, China) using an Omni-ECL™ Ultra-Sensitive Chemiluminescence Detection Kit (Ya Enzyme, Shanghai, China). The antibodies used in this experiment were anti-GAPDH (1:1000, Cell Signaling Technology), anti-CAMK2D (1:1000, Abcam, USA, ab181052), and anti-rabbit IgG (H+L)-HRP (1: 3000; BS13278, Bioworld Technology).
